# Living kidney transplantation without perioperative anticoagulation therapy for a patient with heparin‐induced thrombocytopenia

**DOI:** 10.1002/iju5.12148

**Published:** 2020-02-20

**Authors:** Hayato Nishida, Hiroki Fukuhara, Atsushi Yamagishi, Noriyuki Hosoya, Osamu Ichiyanagi, Toshihiko Sakurai, Sei Naito, Takuya Yamanobe, Tomoyuki Kato, Norihiko Tsuchiya

**Affiliations:** ^1^ Department of Urology Yamagata University Faculty of Medicine Yamagata Japan; ^2^ Department of Urology Tsuruoka Municipal Shonai Hospital Yamagata Japan; ^3^ Department of Urology Yamagata Prefectural Kahoku Hospital Yamagata Japan

**Keywords:** end‐stage renal disease, heparin, heparin‐induced thrombocytopenia, kidney transplantation

## Abstract

**Introduction:**

Heparin‐induced thrombocytopenia is an antibody‐mediated acquired prothrombotic state induced by heparin exposure. The risk of thromboembolic diseases in kidney transplantation with heparin‐induced thrombocytopenia without perioperative anticoagulation has not been determined.

**Case presentation:**

A 64‐year‐old male hemodialysis patient with heparin‐induced thrombocytopenia was referred to our hospital for living kidney transplantation. Anti‐heparin‐induced thrombocytopenia antibody was positive at the time of referral; however, it turned negative 4 months after heparin cessation during hemodialysis sessions. Living kidney transplantation by donation from his wife was performed using the standard technical procedure. Both heparinization and application of medical equipment containing heparin were avoided; however, no anticoagulant was administered intra‐ and postoperatively. The graft kidney functioned immediately, and no thromboembolic event related to heparin‐induced thrombocytopenia occurred.

**Conclusion:**

Kidney transplantation without perioperative anticoagulation therapy after disappearance of anti‐heparin‐induced thrombocytopenia antibody is a well‐tolerated treatment option for patients with end‐stage kidney disease.

**Abbreviations & Acronyms**
Ab: antibody
CT: computed tomography
DFPP: double‐filtration plasmapheresis
DVT: deep vein thrombosis
EIV: external illiac vein
HD: hemodialysis
HIT: heparin‐induced thrombocytopenia
IVC: inferior vena cava
KTx: kidney transplantation
MMF: mycophenolate mofetil
mPSL: methylprednisolone
PE: pulmonary embolism
Tac: tacrolimus
TMA: thrombotic microangiopathy
VTE: venous thromboembolism

**Keynote message**

KTx without perioperative anticoagulation therapy is a well‐tolerated treatment option for end‐stage kidney disease patients with past history of HIT after disappearance of anti‐HIT Ab.

## Introduction

HIT is an Ab‐mediated acquired prothrombotic state induced by heparin exposure. The risk of HIT was reported in approximately 3% of patients who received heparin exposure.^1^ While some cases of HIT in KTx were reported, the risk of thromboembolic diseases in KTx with HIT has not been determined.^2^, ^3^, ^4^, ^5^, ^6^ There were a few case reports on successful KTx in patients with a history of HIT; however, these patients received anticoagulants, although it is not required during typical KTx.^4^, ^5^, ^6^ We report a case of successful living KTx without perioperative anticoagulation in a patient with end‐stage renal disease with a history of HIT.

## Case presentation

**Figure 1:**
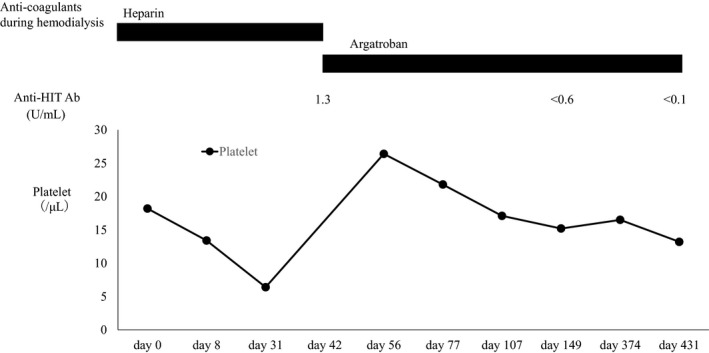
Transition of platelet count and titer of anti‐HIT Ab after HD initiation. The day of HD initiation was set to day 0. The titer of anti‐HIT Ab had been evaluated until day 149 and then examined at our hospital.

A 63‐year‐old male patient with end‐stage renal disease was referred to our hospital for living KTx by donation from his wife. He started HD 2 months before being referred to our hospital. Initially, unfractionated heparin was infused as anticoagulant during HD; however, clotting in the dialysis membrane had frequently occurred, and thrombocytopenia had been gradually exacerbated (Fig. 1). Serum anti‐HIT Ab level was examined for the suspicion of HIT, and seropositivity of anti‐HIT Ab led to the diagnosis of HIT type II. Unfractionated heparin was discontinued and changed to argatroban as the anticoagulant during HD. Thrombocytopenia gradually improved, and events of clotting in the HD membrane also decreased after changing the anticoagulant. Negative conversion of serum anti‐HIT Ab was confirmed 4 months after unfractionated heparin cessation.

**Figure 2:**
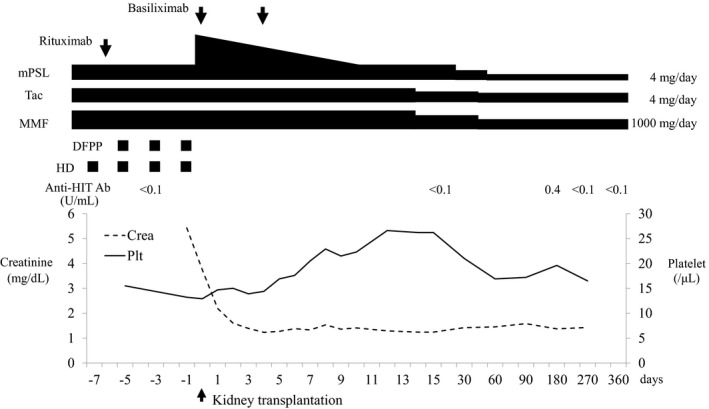
Platelet count, serum creatinine level, and immunosuppressive regimen of the recipient during and after KTx. The day of KTx was set to day 0.

Preservation of seronegative status and absence of thrombotic complications were confirmed by enhanced CT, and then, living KTx between spouses was performed. Maintenance HD therapy and three‐time DFPP against flowcytometry B‐cell crossmatch positivity due to the presence of donor‐specific anti‐HLA Ab (mean fluorescence intensity was 1156) were conducted before KTx using argatroban. Both heparinization and application of medical equipment containing heparin were avoided during the operation. No anticoagulant was administered even during vessel suturing. The graft kidney functioned immediately, and maintenance HD was withdrawn (Fig. 2). No anticoagulant was administered postoperatively. Immunosuppression consisting of steroid, MMF, and Tac and induction therapy consisting of basiliximab and single dose of 200 mg rituximab were adopted for the patient. No thromboembolic adverse event has occurred, and graft function is well‐maintained 1 year after transplantation.

## Discussion

Almost all HD patients are exposed to heparin, which is used as an anticoagulant during each treatment session, and chronic intermittent heparin exposure is associated with developing anti‐heparin Ab, which is observed in approximately 10% of these patients.^7^ Although there is a high prevalence of anti‐heparin Ab in HD patients, thromboembolic complications with thrombocytopenia do not always develop even in this status.^7^ The pretest probability of HIT uses the four *T*’s scoring system: degree of thrombocytopenia, timing of thrombocytopenia with respect to heparin exposure, occurrence of thrombotic complications, and absence of alternative explanations for thrombocytopenia.^5^, ^8^ Our patient did not present with systemic thromboembolic complications but had clotting in the dialysis membrane and more than 50% platelet count fall after heparin exposure without other detectable causes of thrombocytopenia. Both the presence of anti‐heparin Ab with these typical clinical symptoms and recovery of thrombocytopenia after replacement of unfractionated heparin with argatroban support the diagnosis of HIT type II in this case.

We found 10 cases of HIT in KTx^3^, ^4^, ^5^, ^6^, ^9^, ^10^, ^11^, ^12^, ^13^, ^14^ (Table 1). Seven of the 11 patients were diagnosed preoperatively on the basis of the thrombotic complications, while four patients were diagnosed during or after transplantation. All four patients who had not been diagnosed before transplantation and one patient who underwent a successful retransplantation after an initial HIT with graft loss developed thrombotic complications requiring anticoagulation therapy after KTx. Two of the five patients lost graft function due to thrombosis. Six patients diagnosed as having HIT before transplantation did not develop thrombotic complications except for one patient with antiphospholipid Ab syndrome.^11^ The anti‐HIT Ab titer became negative before KTx in all patients except in one who received heart and KTxs. These reports indicate that a history of HIT is not a contraindication for KTx and that the period of negative seroconversion of anti‐HIT Ab may be the preferable time for transplantation. However, all these patients, except our patient, received anticoagulants during the peritransplantation period to minimize the risk of thromboembolic complications due to HIT despite the negative seroconversion of anti‐HIT Ab. Furthermore, we could not find a case of other organ transplantation without anticoagulants during the peritransplantation period.

**Table 1:** Review of literature on KTx with HIT

Organ	Case	Diagnosis of HIT	HIT Ab at KTx	Anticoaglant therapy during operatoin	Thrombotic complication	Author	Year
Kidney	19 years old male	After KTx	N/A	Heparin	TMA in transplanted kidney	Anderegg BA	2005
Kidney	17 years old male	Before KTx	Negative	Recombinant hirudin	None	John U	2006
Kidney	22 years old female	After KTx	N/A	Heparin	Thrombosis from IVC to EIV and PE	Dracopoulos S	2007
Kidney	68 years old male	After KTx	N/A	Heparin	DVT and PE	Maldonado A	2009
Kidney	50 years old male	Before KTx	Negative	Coumadin	DVT and PE	Muzaffar M	2012
Kidney	67 years old female	During KTx	N/A	Dabigatran	DVT	Sakakibara S	2014
Kidney	23 years old female	Before KTx	N/A	Bivalirudin	None	Podolak B	2014
Kidney	64 years old male	Before KTx	Negative	None	None	Our case	2019
Pancreas and kidney	36 years old female	Before KTx	Negative	Dabigatran	None	Jozwik A	2018
Liver and kidney	58 years old male	Before KTx	Negative	Argatroban	None	Taguchi K	2019
Heart and kidney	49 years old female	Before KTx	Positive	Bivalirudin	None	Choxi AA	2017

In patients strongly suspected of having HIT, heparin should be immediately replaced with an appropriate anticoagulant such as argatroban.^15^, ^16^, ^17^ As far as appropriate treatments for HIT are performed, anti‐HIT Ab is transient with a median time of disappearance from 50 to 80 days.^18^ Our patient showed negative conversion of anti‐HIT Ab without life‐threating thromboembolic complications 4 months after heparin cessation and administration of argatroban during HD sessions and then underwent KTx. While we avoided both heparization and application of medical equipment containing heparin during the peritransplant term to minimize the risk of thromboembolic complications due to HIT, we did not administer anticoagulants excluding argatroban during plasmapheresis before transplantation. There were several reasons why we determined the treatment policy. First, anticoagulation during the vessel clamping period in KTx is not usually necessary. Second, clinical episode of thromboembolic complications excluding clot formation in the dialysis membrane had not been observed, and absence of thromboembolic complication was confirmed by systemic enhanced CT examination. A past history of VTE is considered to be the most important risk factor determining VTE recurrence.^19^ KTx without anticoagulants may be considered for recipients who are diagnosed with HIT without systemic thromboembolism. Lastly, non‐heparin anticoagulant was considered to potentially facilitate perioperative bleeding. Argatroban is the only drug approved by Japanese health insurance as an anticoagulant for patients with HIT. No specific antidote is available for argatroban, and thus continuous administration of argatroban during the peritransplant term may make it difficult to control sudden massive bleeding. On the other hand, this is only a single case report. Further investigation including prospective large‐sized clinical studies is required to establish anticoagulant management in KTx of patients with HIT.

We successfully performed living KTx without perioperative anticoagulation in a patient with HIT. KTx without perioperative anticoagulation after disappearance of anti‐HIT Ab could be a well‐tolerated treatment option for patients with end‐stage kidney disease.

## Author contributions

Nishida H contributed to the conception of the work, the acquisition, analysis, and interpretation of data for the work. The author drafted the article. Fukuhara H, Yamagishi A, Hosoya N, Ichiyanagi O, Sakurai T, Naito S, Kawazoe H, Ymanobe T, Kato T, and Tsuchiya N contributed to the analysis and interpretation of data for work. The authors revised the article critically for important intellectual content. All the authors approved the final version of the article to be published. All the authors agreed to be accountable for all aspects of the work in ensuring the questions related to the accuracy or integrity of any part of the work are appropriately investigated and resolved.

## Ethics approval and consent to participate

According to the Ethical Guidelines for Medical and Health Research involving Human Subjects in Japan, ethical approval is not required for case reports.

## Consent for publication

Written informed consent was obtained from the patient for the publication of this case report and any accompanying test results.

## Conflict of interest

The authors declare no conflict of interest.
